# The TOPSHOCK study: Effectiveness of radial shockwave therapy compared to focused shockwave therapy for treating patellar tendinopath - design of a randomised controlled trial

**DOI:** 10.1186/1471-2474-12-229

**Published:** 2011-10-11

**Authors:** Henk van der Worp, Johannes Zwerver, Inge van den Akker-Scheek, Ron L Diercks

**Affiliations:** 1 The Center for Sports Medicine, University Center for Sport, Exercise and Health, University Medical Center Groningen, University of Groningen, Hanzeplein 1, 9700 RB, Groningen, The Netherlands

## Abstract

**Background:**

Patellar tendinopathy is a chronic overuse injury of the patellar tendon that is especially prevalent in people who are involved in jumping activities. Extracorporeal Shockwave Therapy is a relatively new treatment modality for tendinopathies. It seems to be a safe and promising part of the rehabilitation program for patellar tendinopathy. Extracorporeal Shockwave Therapy originally used focused shockwaves. Several years ago a new kind of shockwave therapy was introduced: radial shockwave therapy. Studies that investigate the effectiveness of radial shockwave therapy as treatment for patellar tendinopathy are scarce. Therefore the aim of this study is to compare the effectiveness of focussed shockwave therapy and radial shockwave therapy as treatments for patellar tendinopathy.

**Methods/design:**

The TOPSHOCK study (Tendinopathy Of Patella SHOCKwave) is a two-armed randomised controlled trial in which the effectiveness of focussed shockwave therapy and radial shockwave therapy are directly compared. Outcome assessors and patients are blinded as to which treatment is given. Patients undergo three sessions of either focused shockwave therapy or radial shockwave therapy at 1-week intervals, both in combination with eccentric decline squat training. Follow-up measurements are scheduled just before treatments 2 and 3, and 1, 4, 7 and 12 weeks after the final treatment. The main outcome measure is the Dutch VISA-P questionnaire, which asks for pain, function and sports participation in subjects with patellar tendinopathy. Secondary outcome measures are pain determined with a VAS during ADL, sports and decline squats, rating of subjective improvement and overall satisfaction with the treatment. Patients will also record their sports activities, pain during and after these activities, and concurrent medical treatment on a weekly basis in a web-based diary. Results will be analysed according to the intention-to-treat principle.

**Discussion:**

The TOPSHOCK study is the first randomised controlled trial that directly compares the effectiveness of focused shockwave therapy and radial shockwave therapy, both in combination with eccentric decline squat training, for treating patellar tendinopathy.

**Trial registration:**

Trial registration number NTR2774.

## Background

Patellar tendinopathy (PT) is a chronic overuse injury of the patellar tendon characterised by activity-related anterior knee pain[1]. Actions that require repetitive heavy loading of the knee extensor mechanism as seen in jumping sports like basketball and volleyball are supposed to be the cause, and this is the reason why PT is often referred to as jumper's knee [2]. PT is an injury that can last for years [3]. Several treatment options have been described in the literature, such as rest, anti-inflammatory drugs, physical therapy (eccentric exercises), injections and surgical treatments [4].

Another treatment option for patellar tendinopathy is Extracorporeal Shockwave Therapy (ESWT), a method that was originally used for lithotripsy (kidney stone fragmentation). ESWT uses shockwaves to treat the affected area. A review of the literature concluded that ESWT seems to be a safe and promising treatment for PT, although more research is necessary [5]. The study that showed the most convincing evidence for its effectiveness in treating PT used a combination of ESWT and eccentric training[6]. It seems therefore important to combine these two treatment modalities.

Some years ago shockwave generators were introduced which do not generate focused shockwaves. These generators have a ballistic source that generates radial shockwaves. Cleveland et al. (2007) showed that radial shockwave generators do not generate real shockwaves and that they act only superficially on the tissue [7].

Nowadays radial shockwave generators are often used because they are more affordable than focused shockwave generators. There is however only one study that has investigated the effectiveness of radial shockwave therapy in treating patellar tendinopathy. Lohrer et al. (2002) demonstrated significant effects on pain and function, suggesting that radial shockwave therapy is an effective treatment for patellar tendinopathy [8]. Their study however was non-randomised and had no control group, making it difficult to draw firm conclusions regarding the effectiveness of radial shockwave therapy. Another study by the group directly compared the effectiveness of radial and focused shockwave therapy for treating plantar fasciitis,[9] and that study found a small difference in favour of focused shockwave therapy.

So far no studies have directly compared the effects of focused shockwave therapy and radial shockwave therapy in patellar tendinopathy in one single randomised controlled study. The aim of the TOPSHOCK study is therefore to compare the effectiveness on patellar tendinopathy of both therapies in combination with eccentric decline squat training in a blinded, randomised study.

## Methods/Design

### Design

**Figure 1:**
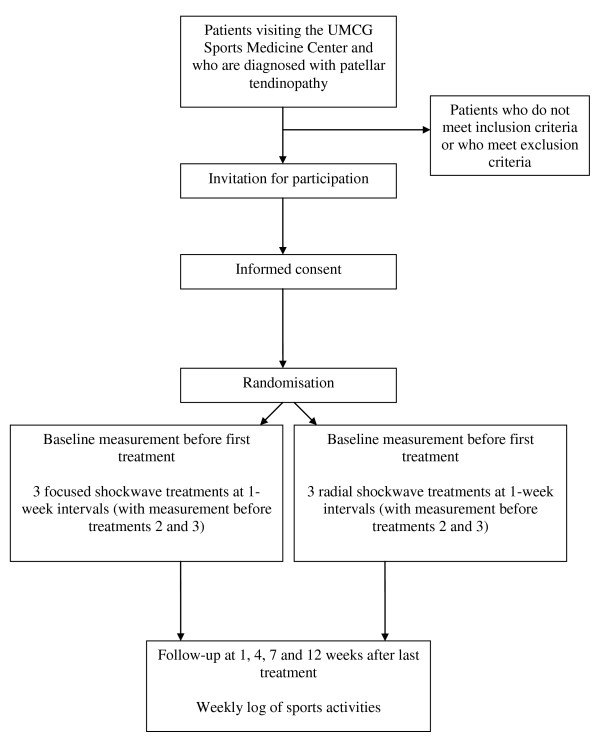
**Flowchart of the TOPSHOCK study**.

The TOPSHOCK study (Tendinopathy Of Patella SHOCKwave) is a two-armed randomised controlled trial. The flow chart of the trial is shown in Figure 1. Patients undergo three sessions of either focused shockwave therapy or radial shockwave therapy in combination with eccentric decline squat training. Outcome assessors and patients are blinded as to which treatment is given. Treatment takes place between June 2010 and November 2011. A baseline measurement will be taken before treatment session 1. Measurements will also take place preceding treatment sessions 2 and 3. Sessions are spaced at 1-week intervals. Follow-up measurements are taken at 1, 4, 7 and 12 weeks after treatment session 3. Two weeks after the final treatment session patients start with a home-based training program consisting of eccentric decline squat training.

The study was approved by the Medical Ethics Committee (Number 2009/322) of the University Medical Center Groningen, The Netherlands. Participants have to provide informed written consent before randomisation.

### Study population

Patients with PT who visit the Center for Sports Medicine of the University Medical Center Groningen and are diagnosed with PT by an experienced sports medicine physician, and who meet the inclusion and exclusion criteria, are invited to participate in the study.

#### Inclusion and exclusion criteria

Patients eligible for inclusion in this study must meet the following criteria: 1. A history of pain in the patellar tendon or its patellar or tibial insertion in connection with training and/or competition; 2. Symptoms for over three months (to exclude acute inflammatory tendon problems and de novo partial ruptures); 3. Age between 18 and 50 (to reduce chances of other osteochondrotic diseases like Sinding-Larsen-Johanson, Osgood-Schlatter and osteoarthrosis); 4. Palpation tenderness of the patellar tendon; 5. A score below 80 on the VISA-P questionnaire (see measurements section) [10,11].

Exclusion criteria for the study are: 1. Acute knee or patellar tendon injuries; 2. Chronic joint diseases; 3. Signs or symptoms of other coexisting knee pathology; 4. Contraindications for ESWT (pregnancy, malignancy, coagulopathy); 5. Knee surgery or injection therapy with corticosteroids in the last three months; 6. Daily use of drugs with a putative effect on patellar tendinopathy in the last year (e.g. non-steroid anti-inflammatory drugs, fluorchinolones), or use of anticoagulants.

### Randomisation

Blockwise randomisation is performed by an independent researcher of another department with a computer-generated randomisation list. This researcher informs the physical therapist who administers the shockwave treatment about the treatment allocation. Patients' allocation will be concealed from the patients and the outcome assessors at all times during the trial.

### Intervention

Both focused and radial shockwave therapy will be given by one experienced independent physical therapist. Shockwave therapy is applied with a shockwave device that has two applicators and can therefore generate both focused and radial shockwaves (Duolith SD1, Storz Medical, Tägerwilen, Switzerland). Both shockwave interventions will be administered without local anaesthesia, since several studies have shown that treatment without anaesthesia is superior to treatment with anaesthesia [12-14].

#### Focused shockwave therapy

Focused shockwave is applied with the focused applicator of the shockwave device. Three sessions will be administered at one-week intervals. Each session consists of 2000 impulses at 4 Hz. The energy flux density is 0.12 mJ/mm^2^, since this corresponds with 2.4 bar on the radial shockwave generator of the used machine (personal communication with manufacturer, 18 May 2010). A fixed energy intensity was chosen instead of a patient guided energy intensity. A patient guided energy intensity may influence the results because one type of shockwave therapy may be more painful compared to the other, therefore leading to a difference in energy intensity level between groups. Rompe et al. (2008) used 2.4 bar for effectively treating insertional Achilles tendinopathy [15], with favourable results and no report of dropouts because of the treatment being to painful. Since PT is often also an insertional tendinopathy the same intensity was chosen in the present study. The patient will be in supine position with the knee slightly flexed. Transmission gel is applied between the applicator and the skin. The applicator is slowly moved around the point of maximal tenderness.

#### Radial shockwave therapy

The procedure for radial shockwave treatment is the same as for focused shockwave therapy, the only difference being that impulses are applied at 8 Hz and the treatment intensity is 2.4 bar.

#### Eccentric decline squat training

Both treatment groups will be given a standard home-based program for tendon training. Peers (2003) showed that combining ESWT with eccentric decline squat training gave positive results [6], therefore this is also applied in the present study. The home program consists of performing squats on a decline board[16]. Three sets of 15 repetitions twice a day for 5 days a week will be prescribed [17]. Performing the exercises takes approximately 10 minutes per day. The physical therapist will give instructions on the home training program during the last treatment session. Patients start with the home program two weeks after the last treatment session and continue until the end of the trial (12 weeks post-treatment). The decline squats should be performed with mild pain (VAS < 4). If pain decreases, load will be increased by adding load in a backpack.

#### Concurrent sports participation and medical treatment

Patients will be allowed to keep on participating in sports activities. If there is an increase in pain in the first 48 hours after treatment the participant will be advised to take paracetamol up to a maximum dose of 3 dd 1000 mg.

### Measurements

#### Baseline measurements

Patients will complete the Dutch VISA-P (Victorian Institute of Sport Assessment) questionnaire at baseline [10,11]. The VISA-P questionnaire is designed to measure severity of patellar tendinopathy. The VISA-P score is the primary outcome variable. Patients will also complete a baseline questionnaire that asks for demographics, sports participation and medical history, knee injuries and previous treatment. Anthropometrics of the participants are also collected. Pain experienced during ADL and sports will be rated on a VAS. Finally, pain during one and during 10 decline squats on a 25° decline board is rated on a VAS.

#### Follow-up measurements

At all follow-up moments (before treatments 2 and 3 and 1, 4, 7 and 12 weeks after treatment) the VISA-P questionnaire is completed. At the 7-week and 12-week follow-up pain VAS score during ADL, sports and functional tests (decline squat) are also collected. At these two moments patients answer a question about subjective improvement and their overall satisfaction with the treatment. A follow-up of 12 weeks was chosen because Peers et al. showed favourable effects of a similar protocol within a 12-week period [6]. Adverse reactions and side effects will also be recorded during the follow-up period.

#### Web-based diary

Every week the patients will record their sports activities, pain during and after these activities, and concurrent medical treatment in a web-based diary.

#### Sample size

Sample size is calculated based on the VISA-P score 12 weeks after treatment. A baseline score of 64 points is expected in symptomatic subjects with an SD of 19 points, based on previous investigations [18]. A difference in the VISA-P score of 15 points at the end of the study (12 weeks) is considered to be clinically relevant. With a power of 80% and an alpha of 5%, 28 tendons per group are needed to detect a difference of 15 points between treatments on the VISA-P questionnaire. Since Peers [6] reported no drop-out of patients because of pain (during or after treatment) in a similar protocol, we do not expect a higher-than-normal drop-out rate.

#### Statistical analyses

Results will be analysed using SPSS version 16 (SPSS, Chicago) according to the intention-to-treat principle. Descriptive statistics will be used to describe the characteristics of the focused and the radial shockwave group and the outcome variables at the evaluation moments. The difference on outcome variables between treatment groups after 12 weeks will be assessed using t-tests. A repeated-measures analysis will be used to determine whether there is a difference on outcome variables between the two groups over time. Analyses will be performed for the primary and secondary variables. A p-value < 0.05 is considered statistically significant.

### Measurements

#### Baseline measurements

Patients will complete the Dutch VISA-P (Victorian Institute of Sport Assessment) questionnaire at baseline [10,11]. The VISA-P questionnaire is designed to measure severity of patellar tendinopathy. The VISA-P score is the primary outcome variable. Patients will also complete a baseline questionnaire that asks for demographics, sports participation and medical history, knee injuries and previous treatment. Anthropometrics of the participants are also collected. Pain experienced during ADL and sports will be rated on a VAS. Finally, pain during one and during 10 decline squats on a 25° decline board is rated on a VAS.

#### Follow-up measurements

At all follow-up moments (before treatments 2 and 3 and 1, 4, 7 and 12 weeks after treatment) the VISA-P questionnaire is completed. At the 7-week and 12-week follow-up pain VAS score during ADL, sports and functional tests (decline squat) are also collected. At these two moments patients answer a question about subjective improvement and their overall satisfaction with the treatment. A follow-up of 12 weeks was chosen because Peers et al. showed favourable effects of a similar protocol within a 12-week period [6]. Adverse reactions and side effects will also be recorded during the follow-up period.

#### Web-based diary

Every week the patients will record their sports activities, pain during and after these activities, and concurrent medical treatment in a web-based diary.

### Sample size

Sample size is calculated based on the VISA-P score 12 weeks after treatment. A baseline score of 64 points is expected in symptomatic subjects with an SD of 19 points, based on previous investigations [18]. A difference in the VISA-P score of 15 points at the end of the study (12 weeks) is considered to be clinically relevant. With a power of 80% and an alpha of 5%, 28 tendons per group are needed to detect a difference of 15 points between treatments on the VISA-P questionnaire. Since Peers [6] reported no drop-out of patients because of pain (during or after treatment) in a similar protocol, we do not expect a higher-than-normal drop-out rate.

### Statistical analyses

Results will be analysed using SPSS version 16 (SPSS, Chicago) according to the intention-to-treat principle. Descriptive statistics will be used to describe the characteristics of the focused and the radial shockwave group and the outcome variables at the evaluation moments. The difference on outcome variables between treatment groups after 12 weeks will be assessed using t-tests. A repeated-measures analysis will be used to determine whether there is a difference on outcome variables between the two groups over time. Analyses will be performed for the primary and secondary variables. A p-value < 0.05 is considered statistically significant.

## Discussion

A number of studies have been conducted on the effect of shockwave therapy for patellar tendinopathy. A review of the literature concluded that ESWT seems to be a safe and promising treatment for PT [5]. All included studies except one used focused shockwave therapy.

Radial shockwave generators generate waves that are very different from those generated by focused shockwave generators. Radial shockwaves lack the characteristic features of shockwaves such as a short rise-time, a high peak pressure and non-linearity [7]. Another difference is that radial shockwaves have a more superficial effect on tissue, compared to focused shockwaves which reach a maximal energy in the focus that is located deeper into the tissue [19]. Since the exact working mechanism of shockwave therapy is not well understood, this difference does not imply that radial shockwave therapy is less effective than focused shockwave therapy [20,21]. Each therapy may even have a different working mechanism [22].

It is not known whether there is a difference in effectiveness between these therapies as treatment for PT. Therefore, the aim is of this study is to directly compare the effects of focused shockwave therapy and radial shockwave therapy on patellar tendinopathy in a blinded randomised controlled trial.

## Conclusions

The TOPSHOCK study is the first blinded randomised controlled trial that directly compares focused shockwave therapy and radial shockwave therapy in combination with eccentric decline squat training for the treatment of patellar tendinopathy.

## List of abbreviations used

TOPSHOCK: Tendinopathy Of Patella SHOCKwave; ESWT: Extracorporeal Shockwave Therapy; VISA-P: Victorian Institute of Sport Assessment - patella

## Competing interests

The authors declare that they do not have competing interest. GymnaUniphy (Berlicum, the Netherlands) supported the TOPSHOCK study by providing the ESWT device. Neither the study nor any of the authors receives or received any funding or financial compensation from GymnaUniphy. GymnaUniphy has not been involved in the design of the study, nor will it be involved in analysis of the data and publications.

## Authors' contributions

HW and JZ conceived of the idea, and developed the intervention. HW wrote the article. IA, HW and JZ developed the design of the trial. JZ recruits the participants and HW is responsible for data acquisition. RD provided advice on the study design. IA, RD and JZ contributed to the content of the article. All authors read and approved the final manuscript.

## Pre-publication history

The pre-publication history for this paper can be accessed here:

http://www.biomedcentral.com/1471-2474/12/229/prepub
